# Supplementary material for: Detection the eDNA of Batrachuperus taibaiensis from the Zhouzhi Heihe River Using a Nested PCR Method and DNA Barcoding
Source: Animals (Basel). 2022 Apr 25;12(9):1105. doi: 10.3390/ani12091105 (PMC9099721; doi:10.3390/ani12091105)
Supplement: Supplementary file 1 [file animals-12-01105-s001.zip › animals-1611466-supplementary.pdf]

Table S1 Total eDNA concentration.

Sampling site of San Cha River				Sampling site of Miao Gou River			
April collection		August collection		April collection		August collection	
Site name	DNA concentration (ng/μl)	Site name	DNA concentration (ng/μl)	Site name	DNA concentration (ng/μl)	Site name	DNA concentration (ng/μl)
4-1-1-1	21.4	8-1-1-1	53.1	4-2-1-1	11.3	8-2-1-1	75.5
	22.3		53.4		12.0		74.7
4-1-1-2	88.6/87.5	8-1-1-2	49.9	4-2-1-2	29.9	8-2-1-2	49.4
4-1-1-3	34.4/35.2		49.5		31.2		49.8
Blank	0.7	Blank	0.7	Blank	0.4	Blank	0.5
4-1-2-1	10.4	8-1-2-1	34.6	4-2-2-1	4.5	8-2-2-1	67.9
	12.5		34.7		5.6		65.9
4-1-2-2	8.4	8-1-2-2	15.5	4-2-2-2	52.4	8-2-2-2	60.4
	9.5		16.5		52.9		61.6
Blank	0.5	Blank	0.6	Blank	0.9	Blank	0.3
4-1-3-1	40.5	8-1-3-1	26.1	4-2-3-1	12.9	8-2-3-1	108.6
	42.5		27.9		13.2		106.4
4-1-3-2	42.2	8-1-3-2	15.8	4-2-3-2	11.9	8-2-3-2	39.5
	43.6		15.9		12.3		38.9
Blank	0.8	Blank	0.2	Blank	0.3	Blank	0.2
4-1-4-1	10.1	8-1-4-1	59.7	4-2-4-1	55.2	8-2-4-1	29.4
	15.1		59.8		59.3		28.3
4-1-4-2	17.1	8-1-4-2	27.0	4-2-4-2	10.1	8-2-4-2	45.4
	18.9		27.1		12.3		45.9
Blank	0.5	Blank	0.1	Blank	0.7	Blank	0.5
4-1-5-1	46.9	8-1-5-1	35.8	4-2-5-1	13.2	8-2-5-1	30.2
	47.6		38.3		14.2		29.9
4-1-5-2	27.8	8-1-5-2	18.2	4-2-5-2	4.4	8-2-5-2	34.2
	29.5		17.8		5.9		35.7
Blank	0.5	Blank	0.5	Blank	0.6	Blank	0.6
4-1-6-1	68.2			4-2-6-1	63		
	69.5				65		
4-1-6-2	37.9			4-2-6-2	28.2		
	38.2				30		
Blank	0.3			Blank	0.6		

Note: “blank” means distilled water was filtered as a negative control for each sample.

Sequences producing significant alignments Download Select columns Show 100

select all *100 sequences selected* GenBank Graphics Distance tree of results MSA Viewe

Description	Scientific Name	Max Score	Total Score	Query Cover	E value	Per. Ident	Acc. Len	Accession
Batrachuperus tibetanus haplotype Hap37 cytochrome b (Cytb) gene, partial cds; mitochondrial	Batrachuperus ti...	195	195	100%	1e-45	98.21%	814	KX548427.1
Batrachuperus tibetanus haplotype Hap36 cytochrome b (Cytb) gene, partial cds; mitochondrial	Batrachuperus ti...	195	195	100%	1e-45	98.21%	814	KX548426.1
Batrachuperus tibetanus haplotype Hap34 cytochrome b (Cytb) gene, partial cds; mitochondrial	Batrachuperus ti...	195	195	100%	1e-45	98.21%	814	KX548424.1
Batrachuperus tibetanus haplotype Hap33 cytochrome b (Cytb) gene, partial cds; mitochondrial	Batrachuperus ti...	195	195	100%	1e-45	98.21%	814	KX548423.1
Batrachuperus tibetanus haplotype Hap32 cytochrome b (Cytb) gene, partial cds; mitochondrial	Batrachuperus ti...	195	195	100%	1e-45	98.21%	814	KX548422.1
Batrachuperus tibetanus haplotype Hap31 cytochrome b (Cytb) gene, partial cds; mitochondrial	Batrachuperus ti...	195	195	100%	1e-45	98.21%	814	KX548421.1
Batrachuperus tibetanus haplotype Hap30 cytochrome b (Cytb) gene, partial cds; mitochondrial	Batrachuperus ti...	195	195	100%	1e-45	98.21%	814	KX548420.1
Batrachuperus tibetanus haplotype Hap28 cytochrome b (Cytb) gene, partial cds; mitochondrial	Batrachuperus ti...	195	195	100%	1e-45	98.21%	814	KX548418.1
Batrachuperus tibetanus haplotype Hap25 cytochrome b (Cytb) gene, partial cds; mitochondrial	Batrachuperus ti...	195	195	100%	1e-45	98.21%	814	KX548415.1
Batrachuperus tibetanus haplotype Hap24 cytochrome b (Cytb) gene, partial cds; mitochondrial	Batrachuperus ti...	195	195	100%	1e-45	98.21%	814	KX548414.1
Batrachuperus tibetanus haplotype Hap23 cytochrome b (Cytb) gene, partial cds; mitochondrial	Batrachuperus ti...	195	195	100%	1e-45	98.21%	814	KX548413.1
Batrachuperus tibetanus haplotype Hap21 cytochrome b (Cytb) gene, partial cds; mitochondrial	Batrachuperus ti...	195	195	100%	1e-45	98.21%	814	KX548411.1
Batrachuperus tibetanus haplotype Hap20 cytochrome b (Cytb) gene, partial cds; mitochondrial	Batrachuperus ti...	195	195	100%	1e-45	98.21%	814	KX548410.1
Batrachuperus tibetanus haplotype Hap19 cytochrome b (Cytb) gene, partial cds; mitochondrial	Batrachuperus ti...	195	195	100%	1e-45	98.21%	814	KX548409.1
Batrachuperus tibetanus haplotype Hap18 cytochrome b (Cytb) gene, partial cds; mitochondrial	Batrachuperus ti...	195	195	100%	1e-45	98.21%	814	KX548408.1
Batrachuperus tibetanus haplotype Hap17 cytochrome b (Cytb) gene, partial cds; mitochondrial	Batrachuperus ti...	195	195	100%	1e-45	98.21%	814	KX548407.1
Batrachuperus tibetanus haplotype Hap10 cytochrome b (Cytb) gene, partial cds; mitochondrial	Batrachuperus ti...	195	195	100%	1e-45	98.21%	814	KX548400.1
Batrachuperus taibaiensis isolate MVZ231041 cytochrome b (cytb) gene, partial cds; mitochondrial	Batrachuperus ta...	195	195	100%	1e-45	98.21%	754	EU296332.1
Batrachuperus taibaiensis strain MVZ231095 cytochrome b gene, partial cds; mitochondrial gene for mitochondri...	Batrachuperus ta...	195	195	100%	1e-45	98.21%	755	AY028758.1
Batrachuperus tibetanus haplotype Hap35 cytochrome b (Cytb) gene, partial cds; mitochondrial	Batrachuperus ti...	189	189	100%	5e-44	97.32%	814	KX548425.1
Batrachuperus tibetanus haplotype Hap29 cytochrome b (Cytb) gene, partial cds; mitochondrial	Batrachuperus ti...	189	189	100%	5e-44	97.32%	814	KX548419.1
Batrachuperus tibetanus haplotype Hap27 cytochrome b (Cytb) gene, partial cds; mitochondrial	Batrachuperus ti...	189	189	100%	5e-44	97.32%	814	KX548417.1
Batrachuperus tibetanus haplotype Hap26 cytochrome b (Cytb) gene, partial cds; mitochondrial	Batrachuperus ti...	189	189	100%	5e-44	97.32%	814	KX548416.1
Batrachuperus taibaiensis isolate MVZ231040 cytochrome b (cytb) gene, partial cds; mitochondrial	Batrachuperus ta...	189	189	100%	5e-44	97.32%	754	EU296331.1
Batrachuperus taibaiensis isolate MVZ231087 cytochrome b (cytb) gene, partial cds; mitochondrial	Batrachuperus ta...	189	189	100%	5e-44	97.32%	754	EU296330.1
Batrachuperus taibaiensis isolate CIB-ZYC524 cytochrome b (cytb) gene, partial cds; mitochondrial	Batrachuperus ta...	183	183	100%	2e-42	96.43%	754	EU296333.1
Batrachuperus taibaiensis isolate MVZ231086 cytochrome b (cytb) gene, partial cds; mitochondrial	Batrachuperus ta...	183	183	100%	2e-42	96.43%	754	EU296329.1
Batrachuperus tibetanus haplotype Hap22 cytochrome b (Cytb) gene, partial cds; mitochondrial	Batrachuperus ti...	178	178	100%	1e-40	95.54%	814	KX548412.1
Batrachuperus tibetanus haplotype Hap14 cytochrome b (Cytb) gene, partial cds; mitochondrial	Batrachuperus ti...	178	178	100%	1e-40	95.54%	814	KX548404.1
Batrachuperus tibetanus haplotype Hap13 cytochrome b (Cytb) gene, partial cds; mitochondrial	Batrachuperus ti...	178	178	100%	1e-40	95.54%	814	KX548403.1
Batrachuperus tibetanus haplotype Hap12 cytochrome b (Cytb) gene, partial cds; mitochondrial	Batrachuperus ti...	178	178	100%	1e-40	95.54%	814	KX548402.1
Batrachuperus tibetanus haplotype Hap11 cytochrome b (Cytb) gene, partial cds; mitochondrial	Batrachuperus ti...	178	178	100%	1e-40	95.54%	814	KX548401.1
Batrachuperus tibetanus haplotype Hap9 cytochrome b (Cytb) gene, partial cds; mitochondrial	Batrachuperus ti...	178	178	100%	1e-40	95.54%	814	KX548399.1
Batrachuperus tibetanus haplotype Hap8 cytochrome b (Cytb) gene, partial cds; mitochondrial	Batrachuperus ti...	178	178	100%	1e-40	95.54%	814	KX548398.1
Batrachuperus tibetanus haplotype Hap7 cytochrome b (Cytb) gene, partial cds; mitochondrial	Batrachuperus ti...	178	178	100%	1e-40	95.54%	814	KX548397.1
Batrachuperus tibetanus haplotype Hap6 cytochrome b (Cytb) gene, partial cds; mitochondrial	Batrachuperus ti...	178	178	100%	1e-40	95.54%	814	KX548396.1
Batrachuperus tibetanus haplotype Hap5 cytochrome b (Cytb) gene, partial cds; mitochondrial	Batrachuperus ti...	178	178	100%	1e-40	95.54%	814	KX548395.1
Batrachuperus tibetanus haplotype Hap4 cytochrome b (Cytb) gene, partial cds; mitochondrial	Batrachuperus ti...	178	178	100%	1e-40	95.54%	814	KX548394.1
Batrachuperus tibetanus haplotype Hap3 cytochrome b (Cytb) gene, partial cds; mitochondrial	Batrachuperus ti...	178	178	100%	1e-40	95.54%	814	KX548393.1
Batrachuperus tibetanus haplotype Hap2 cytochrome b (Cytb) gene, partial cds; mitochondrial	Batrachuperus ti...	178	178	100%	1e-40	95.54%	814	KX548392.1
Batrachuperus tibetanus haplotype Hap1 cytochrome b (Cytb) gene, partial cds; mitochondrial	Batrachuperus ti...	178	178	100%	1e-40	95.54%	814	KX548391.1
Batrachuperus pinchonii voucher BTIBET201411 mitochondrion, complete genome	Batrachuperus pi...	178	178	100%	1e-40	95.54%	16375	KP122337.1
Batrachuperus taibaiensis isolate ROM40082 cytochrome b (cytb) gene, partial cds; mitochondrial	Batrachuperus ta...	178	178	100%	1e-40	95.54%	754	EU296339.1
Batrachuperus taibaiensis isolate ROM40021 cytochrome b (cytb) gene, partial cds; mitochondrial	Batrachuperus ta...	178	178	100%	1e-40	95.54%	754	EU296337.1
Batrachuperus taibaiensis isolate MVZ234925 cytochrome b (cytb) gene, partial cds; mitochondrial	Batrachuperus ta...	178	178	100%	1e-40	95.54%	754	EU296336.1
Batrachuperus taibaiensis isolate MVZ234934 cytochrome b (cytb) gene, partial cds; mitochondrial	Batrachuperus ta...	172	172	100%	5e-39	94.64%	754	EU296342.1
Batrachuperus taibaiensis isolate MVZ234933 cytochrome b (cytb) gene, partial cds; mitochondrial	Batrachuperus ta...	172	172	100%	5e-39	94.64%	754	EU296341.1
Batrachuperus taibaiensis isolate ROM40083 cytochrome b (cytb) gene, partial cds; mitochondrial	Batrachuperus ta...	172	172	100%	5e-39	94.64%	754	EU296340.1
Batrachuperus taibaiensis isolate MVZ234924 cytochrome b (cytb) gene, partial cds; mitochondrial	Batrachuperus ta...	172	172	100%	5e-39	94.64%	754	EU296335.1
Batrachuperus tibetanus isolate CIB-XM1253 cytochrome b (cytb) gene, partial cds; mitochondrial	Batrachuperus ti...	169	169	98%	6e-38	94.50%	745	EU296352.1
Batrachuperus tibetanus isolate CIB-XM1124 cytochrome b (cytb) gene, partial cds; mitochondrial	Batrachuperus ti...	169	169	98%	6e-38	94.50%	744	EU296345.1
Batrachuperus tibetanus isolate CIB-XM1122 cytochrome b (cytb) gene, partial cds; mitochondrial	Batrachuperus ti...	169	169	98%	6e-38	94.50%	754	EU296344.1
Batrachuperus tibetanus strain MVZ231062 cytochrome b gene, partial cds; mitochondrial gene for mitochondrial...	Batrachuperus ti...	169	169	98%	6e-38	94.50%	755	AY028756.1
Batrachuperus tibetanus isolate ROM40034 cytochrome b (cytb) gene, partial cds; mitochondrial	Batrachuperus ti...	163	163	98%	3e-36	93.58%	754	EU296384.1
Batrachuperus tibetanus isolate ROM40033 cytochrome b (cytb) gene, partial cds; mitochondrial	Batrachuperus ti...	163	163	98%	3e-36	93.58%	754	EU296383.1
Batrachuperus tibetanus isolate ROM40024 cytochrome b (cytb) gene, partial cds; mitochondrial	Batrachuperus ti...	163	163	98%	3e-36	93.58%	754	EU296382.1
Batrachuperus tibetanus isolate ROM40023 cytochrome b (cytb) gene, partial cds; mitochondrial	Batrachuperus ti...	163	163	98%	3e-36	93.58%	754	EU296381.1
Batrachuperus tibetanus isolate CIB-XM1258 cytochrome b (cytb) gene, partial cds; mitochondrial	Batrachuperus ti...	163	163	98%	3e-36	93.58%	753	EU296353.1
Batrachuperus tibetanus mitochondrion, complete genome	Batrachuperus ti...	163	163	98%	3e-36	93.58%	16379	DQ333817.1
Batrachuperus tibetanus strain MVZ216665 cytochrome b gene, partial cds; mitochondrial gene for mitochondrial...	Batrachuperus ti...	163	163	98%	3e-36	93.58%	755	AY028772.1
Batrachuperus tibetanus strain MVZ216664 cytochrome b gene, partial cds; mitochondrial gene for mitochondrial...	Batrachuperus ti...	163	163	98%	3e-36	93.58%	755	AY028771.1
Batrachuperus tibetanus strain CIB20118 cytochrome b gene, partial cds; mitochondrial gene for mitochondrial p...	Batrachuperus ti...	163	163	98%	3e-36	93.58%	755	AY028766.1
Batrachuperus tibetanus cytochrome b (CYTB) gene, partial cds; mitochondrial	Batrachuperus ti...	163	163	98%	3e-36	93.58%	1142	AY593144.1
Batrachuperus pinchonii isolate Hap93 cytochrome b (cytb) gene, partial cds; mitochondrial	Batrachuperus pi...	161	161	100%	1e-35	92.86%	764	JQ303937.1
Batrachuperus pinchonii isolate Hap92 cytochrome b (cytb) gene, partial cds; mitochondrial	Batrachuperus pi...	161	161	100%	1e-35	92.86%	764	JQ303936.1
Batrachuperus pinchonii isolate Hap90 cytochrome b (cytb) gene, partial cds; mitochondrial	Batrachuperus pi...	161	161	100%	1e-35	92.86%	764	JQ303934.1
Batrachuperus pinchonii isolate Hap89 cytochrome b (cytb) gene, partial cds; mitochondrial	Batrachuperus pi...	161	161	100%	1e-35	92.86%	764	JQ303933.1
Batrachuperus pinchonii isolate Hap88 cytochrome b (cytb) gene, partial cds; mitochondrial	Batrachuperus pi...	161	161	100%	1e-35	92.86%	764	JQ303932.1
Batrachuperus karlschmidti isolate Hap40 cytochrome b (cytb) gene, partial cds; mitochondrial	Batrachuperus k...	161	161	100%	1e-35	92.86%	764	JQ303884.1
Batrachuperus karlschmidti isolate Hap6 cytochrome b (cytb) gene, partial cds; mitochondrial	Batrachuperus k...	161	161	100%	1e-35	92.86%	764	JQ303850.1
Batrachuperus karlschmidti isolate Hap5 cytochrome b (cytb) gene, partial cds; mitochondrial	Batrachuperus k...	161	161	100%	1e-35	92.86%	764	JQ303849.1
Batrachuperus karlschmidti isolate Hap4 cytochrome b (cytb) gene, partial cds; mitochondrial	Batrachuperus k...	161	161	100%	1e-35	92.86%	764	JQ303848.1
Batrachuperus karlschmidti isolate Hap2 cytochrome b (cytb) gene, partial cds; mitochondrial	Batrachuperus k...	161	161	100%	1e-35	92.86%	764	JQ303846.1
Batrachuperus karlschmidti isolate Hap1 cytochrome b (cytb) gene, partial cds; mitochondrial	Batrachuperus k...	161	161	100%	1e-35	92.86%	764	JQ303845.1
Batrachuperus karlschmidti isolate ROM39976 cytochrome b (cytb) gene, partial cds; mitochondrial	Batrachuperus k...	161	161	100%	1e-35	92.86%	754	EU296317.1
Batrachuperus pinchonii isolate CIB-XM1024 cytochrome b (cytb) gene, partial cds; mitochondrial	Batrachuperus pi...	161	161	100%	1e-35	92.86%	754	EU296269.1
Batrachuperus pinchonii isolate CIB-XM1023 cytochrome b (cytb) gene, partial cds; mitochondrial	Batrachuperus pi...	161	161	100%	1e-35	92.86%	754	EU296267.1
Batrachuperus pinchonii isolate ROM39995 cytochrome b (cytb) gene, partial cds; mitochondrial	Batrachuperus pi...	161	161	100%	1e-35	92.86%	753	EU296261.1
Batrachuperus tibetanus isolate ROM40057 cytochrome b (cytb) gene, partial cds; mitochondrial	Batrachuperus ti...	158	158	98%	1e-34	92.66%	754	EU296368.1
Batrachuperus tibetanus isolate ROM40056 cytochrome b (cytb) gene, partial cds; mitochondrial	Batrachuperus ti...	158	158	98%	1e-34	92.66%	754	EU296367.1
Batrachuperus tibetanus isolate ROM40055 cytochrome b (cytb) gene, partial cds; mitochondrial	Batrachuperus ti...	158	158	98%	1e-34	92.66%	754	EU296366.1
Batrachuperus tibetanus isolate ROM40096 cytochrome b (cytb) gene, partial cds; mitochondrial	Batrachuperus ti...	158	158	98%	1e-34	92.66%	754	EU296356.1
Batrachuperus tibetanus isolate ROM40095 cytochrome b (cytb) gene, partial cds; mitochondrial	Batrachuperus ti...	158	158	98%	1e-34	92.66%	754	EU296355.1
Batrachuperus tibetanus isolate CIB-XM1123 cytochrome b (cytb) gene, partial cds; mitochondrial	Batrachuperus ti...	158	158	98%	1e-34	92.66%	744	EU296347.1
Batrachuperus pinchonii isolate Hap87 cytochrome b (cytb) gene, partial cds; mitochondrial	Batrachuperus pi...	156	156	100%	5e-34	91.96%	764	JQ303931.1
Batrachuperus pinchonii isolate Hap86 cytochrome b (cytb) gene, partial cds; mitochondrial	Batrachuperus pi...	156	156	100%	5e-34	91.96%	764	JQ303930.1
Batrachuperus pinchonii isolate Hap83 cytochrome b (cytb) gene, partial cds; mitochondrial	Batrachuperus pi...	156	156	100%	5e-34	91.96%	764	JQ303927.1
Batrachuperus pinchonii isolate Hap82 cytochrome b (cytb) gene, partial cds; mitochondrial	Batrachuperus pi...	156	156	100%	5e-34	91.96%	764	JQ303926.1
Batrachuperus pinchonii isolate CIB-IOZ3843 cytochrome b (cytb) gene, partial cds; mitochondrial	Batrachuperus pi...	156	156	100%	5e-34	91.96%	754	EU296266.1
Batrachuperus pinchonii isolate CIB-XM905 cytochrome b (cytb) gene, partial cds; mitochondrial	Batrachuperus pi...	156	156	100%	5e-34	91.96%	754	EU296264.1
Batrachuperus pinchonii isolate CIB-XM906 cytochrome b (cytb) gene, partial cds; mitochondrial	Batrachuperus pi...	156	156	100%	5e-34	91.96%	754	EU296256.1
Batrachuperus pinchonii isolate CIB-XM1034 cytochrome b (cytb) gene, partial cds; mitochondrial	Batrachuperus pi...	156	156	100%	5e-34	91.96%	754	EU296254.1
Batrachuperus yenyuanensis strain MVZ231098 cytochrome b gene, partial cds; mitochondrial gene for mitocho...	Batrachuperus y...	156	156	100%	5e-34	91.96%	755	AY028763.1
Batrachuperus yenyuanensis strain MVZ231097 cytochrome b gene, partial cds; mitochondrial gene for mitocho...	Batrachuperus y...	156	156	100%	5e-34	91.96%	755	AY028762.1
Batrachuperus tibetanus haplotype Hap16 cytochrome b (Cytb) gene, partial cds; mitochondrial	Batrachuperus ti...	152	152	98%	7e-33	91.74%	814	KX548406.1
Batrachuperus tibetanus haplotype Hap15 cytochrome b (Cytb) gene, partial cds; mitochondrial	Batrachuperus ti...	152	152	98%	7e-33	91.74%	814	KX548405.1
Batrachuperus pinchonii isolate Hap76 cytochrome b (cytb) gene, partial cds; mitochondrial	Batrachuperus pi...	152	152	98%	7e-33	91.74%	764	JQ303920.1
Batrachuperus londongensis isolate CIB-XM569 cytochrome b (cytb) gene, partial cds; mitochondrial	Batrachuperus lo...	152	152	98%	7e-33	91.74%	754	EU296397.1
Batrachuperus tibetanus isolate ROM40089 cytochrome b (cytb) gene, partial cds; mitochondrial	Batrachuperus ti...	152	152	98%	7e-33	91.74%	754	EU296361.1
Batrachuperus tibetanus isolate ROM40061 cytochrome b (cytb) gene, partial cds; mitochondrial	Batrachuperus ti...	152	152	98%	7e-33	91.74%	754	EU296359.1

Figure S1. List of species identification results using the NCBI GenBank in this study.
